# Short sleep and obesity in midlife and the risk of cognitive decline and incident dementia in later life: the Whitehall II cohort study

**DOI:** 10.12688/wellcomeopenres.23541.1

**Published:** 2025-02-21

**Authors:** Hee Kyung Park, Philipp Frank, Longbing Ren, Gill Livingston, Mika Kivimaki

**Affiliations:** 1 Division of Psychiatry, University College London, London, 6th floor, Wing B, Maple House, W1T 7NF, UK; 2 Institute of Epidemiology and Health Care, University College London, London, England, WC1E 7HB, UK; 3 China center for health development studies, Peking University School of Public Health, Beijing, 100083, China; 4 Camden and Islington NHS Foundation Trust, London, England, UK; 5 Department of Public Health Clinicum, University of Helsinki, Helsinki, 00014, Finland

**Keywords:** dementia, cognition, sleep, obesity, neuroinflammation

## Abstract

Obesity and short sleep duration have both been associated with an increased risk of dementia, but their combined impact and the underlying mechanisms are not yet fully understood. Our aim is to investigate the separate and combined associations of short sleep and obesity with cognitive decline and dementia risk, and to investigate whether these associations are mediated by neuroinflammatory responses and metabolic disturbances, as indicated by blood-based biomarkers. This is a prospective cohort study of adults who were free of dementia, had data on sleep duration and BMI at baseline in 1997-1999, and were tracked for dementia diagnoses until 2023 via linkage to electronic health records. Participants will be divided into four groups: (1) the reference group (2) short sleep (2) short sleep (≤6 hours) and non-obese weight; (3) normal sleep and obesity (≥30kg/m ^2^); (4) short sleep and obesity, the main exposure group. Baseline blood-based biomarkers include glial fibrillary acidic protein (GFAP), chitinase-3-like protein (YKL-40), triggering receptor expressed on myeloid cells 2 (TREM2), neurofilament-light chain (NfL), and brain-derived neurotrophic factor (BDNF), measured from EDTA plasma, as well as insulin resistance measured from fasting serum samples. Cognitive status, measured using tests of executive function, memory, phonemic fluency and semantic fluency, was assessed in 1997-1999, 2002-2004, 2007-2009, 2012-2013, and 2015-2016. We will use linear mixed-effects models and Cox proportional hazard models to examine the associations of short sleep and obesity with change in cognitive functioning and risk of dementia, respectively. To examine whether blood-based biomarkers partially mediate these associations, formal mediation analyses will be performed, estimating the proportion of excess dementia risk mediated by the biomarkers individually and in combination. The results of this study may shed light on the pathomechanisms of sleep and obesity for dementia in terms of neuroinflammation.

## Plain Language Summary

It has been known for some time that a considerable proportion of dementia can theoretically be prevented through controlling of modifiable risk factors, particularly in midlife. There are many postulated risk factors, including short sleep duration and obesity. We will investigate whether short sleep combined with obesity is associated with an increased risk of cognitive impairment in terms of memory, reasoning and fluency, and clinical dementia. If so, we will test what biomarker changes in the body may contribute this as it is unknown. We will use information from an observational cohort study of British civil servants, the Whitehall II study, with a nearly 30 years of follow-up. We will then look at whether inflammation in the brain, as indicated by circulating proteins, is part of the cause. The results of this study may help us understand the importance of normal sleep and weight in terms of brain health.

## Introduction

Dementia significantly impacts individuals, families, and society. The number of people with dementia is rising worldwide as the number of older people increase and in the UK is expected to increase from 870,000 in 2020 to 1.2 million in 2040 ^1^. Therefore, the global burden of dementia sharply increases with age ^2^. A considerable proportion of dementia could potentially be prevented or delayed by addressing modifiable risk factors, although the underlying biological mechanisms linking these risk factors to dementia pathogenesis are not yet fully understood ^3^.

Short sleep duration and obesity are two examples of modifiable risk factors that have been previously linked to an increased risk of dementia. These factors may also play a crucial role in immune system disturbances and metabolic function, such as insulin sensitivity—which may further contribute to dementia risk ^4, 5^. For example, the Framingham Heart study of cognitively unimpaired individuals reported that higher levels of brain-derived neurotrophic factor (BDNF) was associated with a reduced risk for Alzheimer’s disease (AD), and was hypothesized to partially mediate the relationship between lifestyle factors and AD ^6^. Neuroinflammation markers, reflecting reactive astrocytes and activated microglia, include glial fibrillary acidic protein(GFAP), chitinase-3-like protein (YKL-40), and triggering receptor expressed on myeloid cells 2 (TREM2) ^7^. These markers may appear years before the onset of symptoms or deposition of amyloid or tau biomarkers ^8, 9^. Inflammatory cytokines and acute-phase proteins, such as interleukin-6 (IL-6) and high-sensitivity C-reactive protein (hs-CRP), have been found to vary with sleep duration in the Whitehall II study, suggesting a possible relationship between short sleep and acute inflammation ^10^. A recent pilot multi-domain lifestyle intervention study to prevent cognitive decline found plasma measurement of higher levels of BDNF, decreased levels of neuroinflammation markers, including YKL-40 and neurofilament light chain (NfL) in the intervention group showing better cognition compared to controls ^11^.

We will explore whether the combination of short sleep and obesity affects cognition and dementia risk, and whether these associations are partially mediated by blood markers. Specifically, we hypothesize that short sleep + obesity is associated with an increased risk of cognitive decline and incident dementia over a 26-year follow-up period. Furthermore, these associations may be partially mediated through the effects exerted by blood-based neuroinflammatory, neurotrophic, and metabolic markers.

## Methods

This study is reported following the Strengthening the Reporting of Observational Studies in Epidemiology (STROBE) guideline).

The Whitehall II study is an ongoing prospective cohort study of British civil servants, aged 35–55 years, at study entry in 1985–1988 ^12^. A total of 10,308 participants, including 6,895 men and 3,413 women, were asked to complete a series of self-administered questionnaires and attended a baseline screening examination. Questionnaires gathered information on health behaviours, psychosocial factors, and mental health. Follow-up clinical examinations and data collection have taken place every 4 or 5 years.

The present analysis uses data from the Whitehall II study phases 5 (1997–1999, the first phase with assessment of neuroinflammatory, neurotrophic, and metabolic biomarkers), 7 (2002–2004), 9 (2007–2009), 11 (2012–2013), and 12 (2015–2016), as well as linked electronic health records for the diagnosis of dementia until 2023. We will include participants aged ≤65 and free of dementia at baseline (phase 5) with available data on sleep duration, weight, height at baseline, and dementia status at follow-up.

Baseline variables are sleep duration, body mass index (BMI), and demographic variables, including age, sex, ethnicity, marital status, education, family history of dementia, smoking, alcohol, and prevalent health conditions. Sleep duration was assessed using a single item question asking participants about their average weekly sleep duration, with five response categories: ≤5 hours, 6 hours, 7 hours, 8 hours and ≥9 hours. We define short sleep duration as ≤6 hours. Obesity is defined as a BMI of 30kg/m ^2^ or higher. Participants will be divided into four groups: (1) participants with normal sleep (>6 hours and <9 hours) and non-obese weight (<30 kg/m ^2^), the reference; (2) participants with short sleep (≤6 hours) and non-obese weight; (3) participants with normal sleep and obesity (≥30kg/m ^2^); and (4) participants with short sleep and obesity, which is the main exposure in this study.

Primary outcome is diagnosis of incident dementia.

Dementia cases are identified using electronic health records with ICD-10 codes F00-03, F05.1, G30, and G31 through linkage to three national registers- acute hospitals, mental health records and death certificates. Secondary outcome is cognitive decline obtained from repeated clinical examinations, including the Whitehall cognitive test battery which covers fluency, reasoning, and memory from the phase 5, 7, 9, 11, and 12.

Potential mediators, protein concentrations, were assessed at baseline (phase 5). Neuroinflammatory markers include GFAP, YKL-40, and TREM2, and NfL. Inflammatory cytokines include IL-1, IL-1b, IL-1 receptor antagonist (RA), IL-6, tumor necrosis factor (TNF)-α, and hsCRP and anti-inflammatory cytokine, tumor growth factor(TGF)-β1 measured from EDTA plasma. Neurotrophic factor marker is plasma BDNF and metabolic markers include glycaemic and insulin parameters, measured from fasting state serum.

Covariates include age, sex, ethnicity, and available clinical risk factors, all measured at baseline.

## Missing data and statistical outliers

Our analyses will be restricted to participants with complete data on sleep, obesity, and dementia. For mediation analyses, participants will also need to have biomarker data available. For all variables, we report the proportion of missing data. The expected sample size for mediation analyses will be approximately 4,800. Outliers will be identified based on variable distributions.

## Statistical analysis

A two-sided p value of < 0.05 will be considered indicative of statistical significance. To determine whether group 4 (short sleep and obesity) compared to group 1 (normal sleep and no obesity) has a faster cognitive decline, we will use linear mixed-effects models. To investigate whether group 4 is associated with a higher risk of dementia, we will use Cox proportional hazard models, estimating hazard ratios (HRs) and 95% CIs, with group 1 as the reference. In both analyses, we report both minimally and multivariable-adjusted effect estimates.

To examine whether blood-based biomarkers partially mediate these associations, we will conduct formal mediation analyses, estimating the proportion of excess dementia risk mediated by these biomarkers.

## Effect size and statistical power

To calculate the minimal detectable effect size, we used R statistical software (version 4.4.1). Assuming a significance level of α = 0.05, 80% power, a dementia incidence of 10%, 350 participant in the exposure group and 2300 in the reference group, the minimally detectable hazard ratio is around 1.5.

## Sensitivity analysis

We will perform several sensitivity analyses, using an alternative reference group which includes long sleep (≥9 hours) in addition to normal sleep (7–8 hours), an alternative obesity definition based on waist-to-height ratio (≥0.6) instead of BMI, and serial adjustments for a wide range of covariates, including hypertension, diabetes, hyperlipidemia, depression, and physical activity.

## Ethics and consent

Written informed consents were provided by all the participants. The Whitehall II study was approved by the University College London Hospital Committee on the Ethics of Human Research (reference number 85/0938, the approval date 18 August 2022). This study complied with the Declaration of Helsinki ^13^.

## Data Availability

The dataset is available through the application process of data sharing through Dementia Platforms UK (DPUK) (the Whitehall II website, https://www.ucl.ac.uk/psychiatry/research/mental-health-older-people/whitehall-ii/data-sharing). Figshare: STROBE checklist(Table_STROBE.pdf) for " Short sleep and obesity in midlife and the risk of cognitive decline and incident dementia in later life: the Whitehall II cohort study” https://doi.org/10.6084/m9.figshare.28366847.v1 ^14^ Data are available under the terms of the Creative Commons Attribution 4.0 International license (CC-BY 4.0).
