# Supplementary material for: Midlife short sleep with and without obesity, and the risk of future dementia: a prospective cohort study
Source: Wellcome Open Res. 2025 Nov 17;10:91. Originally published 2025 Feb 21. [Version 2] doi: 10.12688/wellcomeopenres.23541.2 (PMC12848887; doi:10.12688/wellcomeopenres.23541.2)
Supplement: Peer review history

# Reviewer report: Reviewer response for version 2

**DOI:** 10.21956/wellcomeopenres.27896.r143945

**Published:** 2026-01-07

**Authors:** David B. Hogan

**Affiliations:** 1 University of Calgary, Calgary, Alberta, Canada

Using data from the Whitehall II study the authors propose to explore whether the combination of short subjective sleep duration (6 hours or less) and a high BMI (30 kg/m ^2^+) at a the time of a mid-life assessment (data derived from the phase 5 visit of the Whitehall II study [1997-1999] when the mean age of included participants was 54.8 years) is associated with the risk of dementia (based on review of electronic health records up to 2023; this is the primary outcome) and/or cognitive decline (based on comparing the cognitive test battery results of phase 5 to results obtained at phases 7 [2002-2004], 9 [2007-2009], 11 [2012-2013], and 12 [2015-2016]), and whether any associations found are at least partially mediated by a number of potential neuroinflammatory, neurotrophic, and metabolic biomarkers. They hypothesize that short subjective sleep duration + a high BMI will be associated with an increased risk of incident dementia and cognitive decline. Covariates in their core analyses would include age, sex (women, men), ethnicity (white, non-white), and 10/13 dementia risk factors suggested by the Lancet Standing Commission.

 The protocol was clearly written other than for a number of errors in the abstract that would be easy to correct (e.g., "duration", "an well-known", "short sleep obesity"). I did have a number of comments to share that I hope might lead to improvements in the protocol:

 1.  I realize the authors are limited to the data that was collected on Whitehall II participants but their data on sleep duration is based on the response to a single question on the participant's estimate of average sleep duration on week nights. Doubts have been raised the validity of subjective estimates of sleep duration. These estimates do not appear to correlate well with objective measures of sleep duration. This concern might come up as a limitation to any conclusions they might draw from the results of their proposed work.

 2. The Whitehall II study did ask a question about the frequency of certain sleep problems (i.e., trouble falling asleep, waking up several times during the night, trouble staying asleep, waking up feeling tired/ worn out). It might be worthwhile to look at the responses to this question to get a sense of sleep quality experienced by participants and to determine whether this variable was associated with their outcomes  of interest (incident dementia, cognitive decline).

 3. The lack of any information on whether participants might have a sleep disorder like Obstructive Sleep Apnea (OSA) would be another limitation. For example, OSA is associated with being overweight/obese, altered sleep duration (may be shortened as a result of frequent arousals or prolonged as compensation for poor sleep quality), and is a significant risk factor for dementia. OSA could potentially explain an association between sleep duration, obesity, and dementia risk. Again, this might come up when study limitations are enumerated.

 4. I'm sure the authors are aware that BMI is falling out of favour as a measure of obesity and predictor of adverse health risk particularly in older and more muscular adults. Other measures such as the waist-to-height ratio might be more predictive. Waist circumference was collected in the Whitehall II study, and I see will be examined in their sensitivity analysis. I think the limitations of BMI would suggest to me that waist-to-height ratio should be their primary obesity measure and not BMI (which could be examined in their proposed sensitivity analysis). I'd suggest switching how BMI and waist-to-height ratio will be used in the protocol.

 5. From their Methods, I understand APOE status will be determined, but I'm unclear exactly how they intend to use this information in their analyses.

 6. For clarification, I'd ask the authors to comment on why they excluded participants who reported sleeping 9+ hours in their core analyses (I appreciate they will be examined in their planned sensitivity analysis). I can think of reasons for this but would ask the authors to explicitly state why.

 7. How comprehensive do they believe dementia identification was in the electronic health records they used to identify its development? Administrative health records tend to underestimate the presence of dementia (especially milder cases) from my reading of the literature. 

 8. More information should be provided on the cognitive test battery used in the study. What specific tests were done? Did they remain consistent across phases 5, 7, 9, 11, and 12? Do they plan to look at each test individually or are they planning to assess overall performance over time? If the latter, how will they assess overall performance? What normative data will they use? Will they be correcting for age +/- sex and education?

 9. The analytical sample was stated to be 4,769 participants. Is this the number at the time of phase 5? If so, do the authors know the degree of attrition of this initial sample at the time of the follow-up phases? This would be particularly important for their secondary outcome of cognitive decline. 

 10. Lastly, do the authors know how consistent across phases were the BMI and sleep duration measures? I'd be particularly interested in comparing phase 5 with 7 results.

Is the study design appropriate for the research question?

Partly

Is the rationale for, and objectives of, the study clearly described?

Yes

Are sufficient details of the methods provided to allow replication by others?

Partly

Are the datasets clearly presented in a useable and accessible format?

Partly

Reviewer Expertise:

Geriatric medicine, cognitive decline and dementia in later life

I confirm that I have read this submission and believe that I have an appropriate level of expertise to confirm that it is of an acceptable scientific standard, however I have significant reservations, as outlined above.

# Reviewer report: Reviewer response for version 2

**DOI:** 10.21956/wellcomeopenres.27896.r139907

**Published:** 2026-01-05

**Authors:** Mahsa Dadar

**Affiliations:** 1 McGill University, Montreal, Québec, Canada

No further comments.

Is the study design appropriate for the research question?

Partly

Is the rationale for, and objectives of, the study clearly described?

Yes

Are sufficient details of the methods provided to allow replication by others?

Partly

Are the datasets clearly presented in a useable and accessible format?

Not applicable

Reviewer Expertise:

Cerebrovascular disease, neurodegenerative disease, imaging, computational modelling

I confirm that I have read this submission and believe that I have an appropriate level of expertise to confirm that it is of an acceptable scientific standard.

# Reviewer report: Reviewer response for version 2

**DOI:** 10.21956/wellcomeopenres.27896.r139906

**Published:** 2026-01-05

**Authors:** Atticus H Hainsworth

**Affiliations:** 1 St George's University of London Neuroscience Research Centre (Ringgold ID: 200478), London, UK

My comments on the original submission have not been addressed adequately. Changes to my earlier report are marked **** below.

 This is a study protocol for a post-hoc analysis within the Whitehall-2 study of UK civil servants. 

 ... I think I am correct that this is a retrospective study. The data are already on file, and the hypothesis is being tested post-hoc. This should be made clear in the abstract. ***** This has not been adequately addressed. The word "prospective" has even been added to the title! If all the data are on file before you start the study, it is not a prospective study. Am I missing something?

 Other points

 The title seems long and rather cumbersome. And it does not mention the blood biomarkers ***** title should include the term "blood biomarkers". It should also included the word "protocol". E.g. Midlife short sleep with and without obesity, and the risk of future dementia: a protocol for a cohort study of biomarkers

 The Abstract should give some basic demographic information about the cohort. N number, proportion males and females, average age, fraction of non-whites. ***** this has not been addressed

 It would not hurt for the text to have an English and grammar check. ***** The Abstract still reads awkwardly. Some typos have been added for good measure (e.g. leep not sleep)

Is the study design appropriate for the research question?

Partly

Is the rationale for, and objectives of, the study clearly described?

Yes

Are sufficient details of the methods provided to allow replication by others?

Partly

Are the datasets clearly presented in a useable and accessible format?

Not applicable

Reviewer Expertise:

Vascular causes of dementia; cerebral small vessel disease

I confirm that I have read this submission and believe that I have an appropriate level of expertise to confirm that it is of an acceptable scientific standard, however I have significant reservations, as outlined above.

# Reviewer report: Reviewer response for version 1

**DOI:** 10.21956/wellcomeopenres.25966.r127720

**Published:** 2025-08-29

**Authors:** Mahsa Dadar

**Affiliations:** 1 McGill University, Montreal, Québec, Canada

The study protocol proposes a retrospective analysis of the Whitehall II data. The proposed analyses are in general appropriate for the hypotheses of interest, however, there are some details that require further clarification/justification. Here are my main comments/suggestions:

 - The authors should provide baseline demographics details on the study participants.

 - Were any of the included participants underweight (BMI < 18.5)?  Since underweight is also associated with increased health risks and mortality, if yes, this group should be assessed separately or excluded (if the sample size is too small).

 - Will any exclusion criteria be applied with respect to other risk factors and disorders associated with dementia (e.g. diabetes, hypertension, history of strokes, presence of neurological conditions, etc.), please provide details. 

 - "We will include participants aged ≤65 and free of dementia at baseline". If individuals with MCI at baseline are included in the analyses, the percentage of MCI participants at baseline in each group should be clarified, and baseline cognition should also be included as a covariate in all analyses.  

 - Please provide details on the "available clinical risk factors" that will be included as covariates in the analyses, and whether the groups of interest are different with respect to these risk factors.

 - Will the missing co-variables be imputed? please provide details.

 - Effect size and Statistical Power: what was the dementia incidence rate of 10% assumption based on? Is that a reasonable estimate for the age range of the included participants? please provide references.

Is the study design appropriate for the research question?

Partly

Is the rationale for, and objectives of, the study clearly described?

Yes

Are sufficient details of the methods provided to allow replication by others?

Partly

Are the datasets clearly presented in a useable and accessible format?

Not applicable

Reviewer Expertise:

Cerebrovascular disease, neurodegenerative disease, imaging, computational modelling

I confirm that I have read this submission and believe that I have an appropriate level of expertise to confirm that it is of an acceptable scientific standard, however I have significant reservations, as outlined above.

# Response

**Published:** 2025-11-09

**Authors:** Hee Park

**Affiliations:** Psychiatry, Not Applicable, London, UK

We would like to thank the reviewers for their thoughtful and constructive feedback on our protocol manuscript. We have carefully addressed each point raised, providing additional methodological details, revisions to the abstract and title, and clarification of the analytical approach where appropriate.

**REVIEWER COMMENT:** The study protocol proposes a retrospective analysis of the Whitehall II data. The proposed analyses are in general appropriate for the hypotheses of interest, however, there are some details that require further clarification/justification. Here are my main comments/suggestions:

 1. The authors should provide baseline demographics details on the study participants. **Authors’ response:** We thank the reviewer for this helpful comment. Baseline demographics have now been added as follows. This analysis is based on the British Whitehall II study and the final analytical sample included 4,769 participants after 1,782 excluding participants. In this predominantly white cohort (91.9%), the mean age at baseline (1997-1999) was 54.8 years (standard deviation [SD], 5.4), and 29.0% of the participants were women. The mean follow-up duration was 23.0 years (SD, 4.7).

 2. Were any of the included participants underweight (BMI <18.5)? Since underweight is also associated with increased health risks and mortality, if yes, this groups should be assessed separately or excluded (if the sample size is too small).

**Authors’ response:** Thank you for raising this point. Only 39 participants met the underweight criterion (mean BMI =17.54, SD=0.84). Given the very small sample size, they were unlikely to influence results, we therefore did not plan to conduct separate analyses for this group; they were retained in the dataset and classified within the non-obesity group.

 3. Will any exclusion criteria be applied with respect to other risk factors and disorders associated with dementia (e.g. diabetes, hypertension, history of strokes, presence of neurological conditions, etc.), please provide details.

**Authors’ response:** We have not excluded other risk factors associated with dementia. We have clarified the exclusion criteria as follows.Participants were excluded if they were ≥65 years old (n=547); had missing data on sleep and obesity or sleep duration 9 hours, n=993) or mediators (blood proteins; n=227); or developed dementia within the first 10 years of follow-up (n=15). Other potential risk factors, including vascular factors, were treated as covariates in the sensitivity analyses, as detailed below. Covariates include age, sex, ethnicity, and available clinical risk factors, all measured at baseline. Age was treated as a continuous variable. Sex (men and women) and Ethnicity (white and non-white) was treated as a binary variable, white and non-white. For sensitivity analysis, baseline global cognitive score was included as an additional covariate. Apolipoprotein E (APOE) genotype was treated as a categorical variable (no, one or two APOE 4 alleles). Obesity was the exposure of interest and 10 of the other 13 risk factors suggested by the Lancet Standing Commission3 were available: hypertension, diabetes, high LDL cholesterol, depression, hearing impairment, social isolation, physical inactivity, smoking, heavy alcohol, and less education (<9 years) , all used as a categorical variable (absence or presence).

 4. “We will include participants ages 65 and free of dementia at baseline.” If individuals with MCI at baseline are included in the analyses, the percentage of MCI participants at baseline in each group should be clarified, and baseline cognition should also be included as a covariate in all analyses.

**Authors’ response:** We appreciate the reviewer’s insightful comments. The study design included data on dementia diagnoses obtained from electronic health records, but not on mild cognitive impairment (MCI). Therefore, the proportion of participants with MCI could not be determined. However, the baseline cognitive score was lower in people with short sleep and obesity (mean z score [SD]: -0.15 [1.02], p<0.01) compared with other groups. Baseline cognition was included as a covariate in the sensitivity analyses.

 5. Please provide details on the “available clinical risk factors” that will be included as covariates in the analyses, and whether the groups of interest are different with respect to these risk factors.

**Authors’ response: **Thank you for the comment. As described in our response to Question 3, detailed covariates are underlined in the following paragraph. Differences between exposure groups will be reported in the baseline characteristics table in the final manuscript following completion of the analyses. Covariates include age, sex, ethnicity, and available clinical risk factors, all measured at baseline. Age was treated as a continuous variable. Sex (men and women) and Ethnicity (white and non-white) was treated as a binary variable, white and non-white. For sensitivity analysis, baseline global cognitive score was included as an additional covariate. Apolipoprotein E (APOE) genotype was treated as a categorical variable (no, one or twoAPOE 4 alleles). Obesity was the exposure of interest and 10 of the other 13 risk factors suggested by the Lancet Standing Commission3 were available: hypertension, diabetes, high LDL cholesterol, depression, hearing impairment, social isolation, physical inactivity, smoking, heavy alcohol, and less education (<9 years) , all used as a categorical variable (absence or presence).

 6. Will the missing co-variables be imputed? Please provide details.

**Authors’ response:** Thank you for raising this point. The number and percentage of the missing co-variables will be presented in the manuscript after completion of the statistical analyses. No imputation will not be performed.

 7. Effect size and Statistical Power: What was the dementia incidence rate of 10% assumption based on? Is that a reasonable estimate for the age range of the included participants? Please provide references.

**Authors’ response:** We appreciate the important point. In our study, both exposures− short sleep and obesity − may be midlife risk factors for dementia. Short sleep is a putative risk for dementia, whereas midlife obesity is an established risk factor for dementia. In a large-scale, population-based study (n=1,229; the mean follow-up period=21 (4.9) years), the incidence of dementia of people with midlife obesity (BMI >30) was 9.5% (21/222) (Obesity and Vascular Risk Factors at Midlife and the Risk of Dementia and Alzheimer Disease. Arch Neurol. 2005;62(10):1556–1560.) In another longitudinal, population-based study using the Cardiovascular Risk Factors, Aging, and Dementia (CAIDE) score, representing midlife risk score, the risk of dementia was 7.4% for a 10-11 CAIDE score and 16.5% for a 12-15 CAIDE score. (Risk score for the prediction of dementia risk in 20 years among middle aged people: a longitudinal, population-based study. Lancet Neurol. 2006 Sep;5(9):735-41.) The results of these study were used in selecting participants of the Finnish Geriatric Intervention Study to Prevent Cognitive Impairment and Disability (FINGER) study. (The Finnish Geriatric Intervention Study to Prevent Cognitive Impairment and Disability (FINGER): study design and progress. Alzheimers Dement. 2013 Nov;9(6):657-65.) Based on these findings, we conservatively assumed an approximate 10% dementia incidence among midlife individuals with risk factors such as short sleep and obesity.

# Reviewer report: Reviewer response for version 1

**DOI:** 10.21956/wellcomeopenres.25966.r126457

**Published:** 2025-08-12

**Authors:** Atticus H Hainsworth

**Affiliations:** 1 St George's University of London Neuroscience Research Centre (Ringgold ID: 200478), London, UK

This is a study protocol for a post-hoc analysis within the Whitehall-2 study of UK civil servants. It is in general well explained. A few aspects need clarification.

 I have only two conceptual issues.

 First, I think I am correct that this is a retrospective study. The data are already on file, and the hypothesis is being tested post-hoc. This should be made clear in the abstract.

 Second, the authors appear to believe that GFAP, YKL40, NFL and sundry others are markers of inflammation, or of neuroinflammation. This is incorrect and a bit misleading. It might be better in the Abstract simply to list them, in addition to the established markers of inflammation (IL-1, TNF, CRP et cetera).

 Other points

 The title seems long and rather cumbersome. And it does not mention the blood biomarkers

 The Abstract should give some basic demographic information about the cohort. N number, proportion males and females, average age, fraction of non-whites.

 Methods should give some indication of how the various blood markers were assayed. What platform? How the samples stored? What %CV is considered acceptable?

 It would not hurt for the text to have an English and grammar check.

Is the study design appropriate for the research question?

Partly

Is the rationale for, and objectives of, the study clearly described?

Yes

Are sufficient details of the methods provided to allow replication by others?

Partly

Are the datasets clearly presented in a useable and accessible format?

Not applicable

Reviewer Expertise:

Vascular causes of dementia; cerebral small vessel disease

I confirm that I have read this submission and believe that I have an appropriate level of expertise to confirm that it is of an acceptable scientific standard, however I have significant reservations, as outlined above.

# Response

**Published:** 2025-11-09

**Authors:** Hee Park

**Affiliations:** Psychiatry, Not Applicable, London, UK

We would like to thank the reviewers for their thoughtful and constructive feedback on our protocol manuscript. We have carefully addressed each point raised, providing additional methodological details, revisions to the abstract and title, and clarification of the analytical approach where appropriate.

**REVIEWER COOMENT:** This is a study protocol for a post-hoc analysis within the Whitehall II study of UK civil servants. It is in general well explained. A few aspects need clarification. I have only two conceptual issues.

 1. First, I think I am correct that this is a retrospective study. The data are already on file, and the hypothesis is being tested post-hoc. This should be made clear in the abstract.

**Authors’ response:** Thank you for your comment. The data was collected in prospective waves, so we do not agree. We agree and have revised the Abstract to clarify the study design. This is an analysis of a prospective cohort study of adults who were free of dementia, had data on sleep duration and BMI at baseline in 1997-1999, and were prospectively tracked for dementia diagnoses until 2023 via linkage to electronic health records.

 2. Second, the authors appear to believe that GFAP, YKL-40, NfL and sundry others are markers of inflammation, or of neuroinflammation. This is incorrect and a bit misleading. It might be better in the Abstract simply to list them, in addition to the established markers of inflammation (IL-1, TNF, CRP, etc.)

**Authors’ response: **Thank you for raising this point. We have revised the abstract to reflect this suggestion. Baseline blood-based biomarkers include chitinase-3-like protein (YKL-40), triggering receptor expressed on myeloid cells 2 (TREM2), neurofilament-light chain (NfL), brain- derived neurotrophic factor (BDNF), interleukin (IL)-1, IL-6, C-reactive protein (CRP), transforming growth factor (TGF)-3, adiponectin, insulin, ghrelin, and leptin.

 3. The title seems long and rather cumbersome. And it does not mention the blood biomarkers.

**Authors’ response:** Thank you for the comments. The title has been revised to: Midlife short sleep with and without obesity, and the risk of future dementia: a prospective cohort study

 4. Methods should give some indication of how the various blood markers were assayed. What platform? How the sample stored? What %CV is considered acceptable?

**Authors’ response: **We have added methodological details as follows: Potential mediators, protein concentrations, were assessed at baseline (phase 5). Blood samples were immediately frozen at -80C and were analysed using the SOMAscan version 4 assay, which has a median intra- and inter-assay coefficients of variation (CV) of approximately 5%.13 Neuroinflammatory markers include YKL-40, and TREM2, and NfL. Pro- inflammatory markers, including IL-1, IL-6, and hsCRP, as well as anti-inflammatory cytokine, transforming growth factor(TGF)-1, were obtained from EDTA plasma. Neurotrophic factor marker is plasma BDNF and metabolic markers include adiponectin, insulin, ghrelin, and leptin, measured from fasting state serum. 13. Candia J, Cheung F, Kotliarov Y, et al. Assessment of Variability in the SOMAscan Assay. Sci Rep. 2017 Oct 27;7(1):14248.

 5. It would not hurt for the text to have an English and grammer check.

**Authors’ response:** We thank the reviewer for this helpful suggestion. The manuscript has been thoroughly reviewed, and the English language and grammar have been corrected throughout to ensure clarity and readability.
